# Apolipoprotein C-II Mimetic Peptide Promotes the Plasma Clearance of Triglyceride-Rich Lipid Emulsion and the Incorporation of Fatty Acids into Peripheral Tissues of Mice

**DOI:** 10.1155/2019/7078241

**Published:** 2019-02-03

**Authors:** Tomohiro Komatsu, Toshihiro Sakurai, Anna Wolska, Marcelo J. Amar, Akiko Sakurai, Boris L. Vaisman, Denis Sviridov, Stephen Demosky, Milton Pryor, Katsunori Ikewaki, Alan T. Remaley

**Affiliations:** ^1^Lipoprotein Metabolism Laboratory, Translational Vascular Medicine Branch, National Heart, Lung, and Blood Institute, National Institutes of Health, Bethesda, Maryland, USA; ^2^Division of Anti-aging and Vascular Medicine, Department of Internal Medicine, National Defense Medical College, Tokorozawa, Japan; ^3^Faculty of Health Sciences, Hokkaido University, Sapporo, Japan

## Abstract

**Aim:**

Plasma apolipoprotein C-II (apoC-II) activates lipoprotein lipase (LPL) and thus lowers plasma triglycerides (TG). We previously reported that a human apoC-II mimetic peptide (C-II-a) decreased plasma TG in apoC-II mutant mice, as well as in apoE-knockout mice. Because it is unknown what tissues take up free fatty acids (FFAs) released from TG after C-II-a peptide administration, we investigated in mice TG plasma clearance and tissue incorporation, using ^3^H-triolein as a tracer, with and without C-II-a treatment.

**Methods and Results:**

Intralipid® fat emulsion was labeled with ^3^H-triolein and then mixed with or without C-II-a. Addition of the peptide did not alter mean particle size of the lipid emulsion particles (298 nm) but accelerated their plasma clearance. After intravenous injection into C57BL/6N mice, the plasma half-life of the ^3^H-triolein for control and C-II-a treated emulsions was 18.3 ± 2.2 min and 14.8 ± 0.1 min, respectively. In apoC-II mutant mice, the plasma half-life of ^3^H-triolein for injected control and C-II-a treated emulsions was 30.1 ± 0.1 min and 14.8 ± 0.1 min, respectively. C57BL/6N and apoC-II mutant mice at 120 minutes after the injection showed increased tissue incorporation of radioactivity in white adipose tissue when C-II-a treated emulsion was used. Higher radiolabeled uptake of lipids from C-II-a treated emulsion was also observed in the skeletal muscle of C57BL/6N mice only. In case of apoC-II mutant mice, decreased uptake of radioactive lipids was observed in the liver and kidney after addition of C-II-a to the lipid emulsion.

**Conclusions:**

C-II-a peptide promotes the plasma clearance of TG-rich lipid emulsions in wild type and apoC-II mutant mice and promotes the incorporation of fatty acids from TG in the lipid emulsions into specific peripheral tissues.

## 1. Introduction

Plasma triglycerides (TG) are major energy source for peripheral tissues and are primarily transported either in large chylomicron particles, which are formed in the intestine after a meal or in smaller hepatic derived very low density lipoprotein (VLDL) particles, which are eventually transformed by the lipolysis of TG into LDL particles [1]. Hypertriglyceridemia (HTG), particularly when associated with partially lipolyzed remnant particles, is now recognized to be a major risk factor for cardiovascular disease [2–4]. In contrast, the markedly elevated level of plasma TG that occurs in Type I hyperlipidemia, when chylomicron particles are too large to enter the vessel wall, is instead associated with pancreatitis and hepatosplenomegaly [5].

HTG is most commonly due to polygenic and dietary factors and is often present in patients with obesity [6], metabolic syndrome [7], and diabetes mellitus [4], which are all conditions associated with cardiovascular disease from increased remnant particles. When HTG is caused by rare monogenetic mutations in genes like lipoprotein lipase (LPL) [8], the main enzyme that hydrolyzes plasma TG, or apolipoprotein C-II (apoC-II) [9], an activator of LPL, larger lipoprotein TG-rich particles tend to accumulate and these patients are at a risk for pancreatitis [10].

LPL is mainly synthesized in adipose tissue, muscles, and is transported to the luminal side of vascular endothelium where lipoproteins are located [11]. LPL hydrolyzes triglycerides in TG-rich lipoproteins, such as chylomicrons and VLDL, to diglycerides, monoglycerides, and fatty acids (FFAs). The released FFAs are quickly taken up by peripheral tissues by specific transporters or by passive diffusion, where they either undergo beta oxidation for energy production or are stored in lipid droplets after re-esterification into TG [1, 11]. Human apoC-II, a 79-amino acid long apolipoprotein, is a key cofactor for LPL activation [12, 13]. Very high levels of apoC-II, however, can inhibit *in vitro* LPL activity [14], as has been observed in apoC-II transgenic mice with HTG [15].

We recently described a novel bihelical human apoC-II mimetic peptide called C-II-a [16]. It contains an artificial amphipathic helix for anchoring to lipoproteins and a second helix based on the C-terminal domain of apoC-II, which is known to activate LPL. The peptide is equally potent to full-length human apoC-II in activating LPL. It also enhances lipolysis in an *ex vivo* plasma assays from not only patients with apoC-II deficiency [16, 17] but also from patients with other forms of HTG [1, 16, 17]. *In vivo*, the peptide was shown to markedly and rapidly lower TG in apoC-II mutant mice [18]. Similarly, the peptide was also found to lower TG in apoC-II-KO zebrafish [19] and in apoE-KO mice [16].

Several animal studies have reported tissue FFA distribution after TG lipolysis from infused fat emulsion containing radiolabeled TG [20, 21]. High affinity uptake of FFAs and TG metabolites was observed in many tissues, but most notably in the liver, adipose tissue, skeletal muscle, and spleen. Because apoC-II mimetic peptides could be a potential therapy for apoC-II deficiency, as well as possibly for other forms of HTG, it is important to establish in what tissues it promotes FFAs and TG metabolite uptake. Such findings may also be relevant for the use of apoC-II mimetic peptides in modulating TG metabolism in patients given total parenteral nutrition containing TG-rich lipid emulsions. Here, we, therefore, investigated the effect of the C-II-a peptide on TG lipolysis and metabolite uptake in peripheral tissues in wild type mice and apoC-II mutant mice intravenously injected with TG-rich lipid emulsions.

## 2. Methods

### 2.1. Lipid Emulsion Preparation

Intralipid® 20%, (Fresenius Kabi, Uppsala, Sweden), which contains 20% soybean oil, 1.2% egg yolk phospholipids, and 2.25% glycerin, was used as the source of the TG-rich lipid emulsion. Triolein, [9,10-^3^H(N)] (Perkin Elmer, Boston, USA, 0.5 mCi/ml) in toluene was evaporated under N_2_ gas and served as the source of radiolabeled TG. Intralipid was mixed by gentle stirring with triolein, [9,10-^3^H(N)] at 4°C overnight. Reisolation of the TG-rich lipid emulsion by density gradient ultracentrifugation showed that approximately 91% of the radiotracer was associated with particles with a density of <0.95 g/mL. Each mouse was injected with a dose 4.2 *µ*l of labeled lipid emulsion per 1 g of body weight, which corresponds to the same concentration of Intralipid used in humans. Each dose contained approximately 1,100–1,900 CPM/mg mouse body weight of ^3^H-radioactive counts from the radiolabeled fatty acids in the triolein.

The C-II-a peptide dissolved in saline was synthesized as previously described [16]. It was gently mixed with Intralipid for 5 min at room temperature just before intravenous injection to yield a final concentration of 0.775 mmol C-II-a per 1 ml of solution, which is a dose of the peptide that yields the maximum LPL activation effect based on previous *in vitro* activity assays and *in vivo* animal studies [16, 17]. Lipid emulsion treated with only the saline vehicle was similarly prepared as a control. The mean particle size of the two different lipid emulsions was determined by dynamic light scattering (Beckman Coulter, CA, USA). Reisolation of the lipid emulsion by density gradient ultracentrifugation (<0.95 g/mL) after incubation with TAMRA-labeled C-II-a peptide showed that 90% of the peptide was incorporated into the emulsion particles after the 5 min incubation period. C-II-a was labeled with TAMRA on the N-terminus after synthesis by standard FMOC-chemistry and purified to greater than 95% homogeneity by C-18 reverse phase HPLC.

### 2.2. LPL Activity Assays

LPL activity assays were performed as previously described [16]. Briefly, Intralipid (160 *µ*g TG per well) served as the substrate in a reaction volume of 50 *µ*l in phosphate-buffered saline (pH 7.4), containing 0.1% (w/v) fatty acid free bovine serum albumin, and the generation of FFAs after 1 h at room temperature was used as a measure of LPL activity [22]. A total of 0.2 units of LPL purified from bovine milk (Sigma-Aldrich, St. Louis, MO) was added as well. FFAs generated during the reaction were quantified in the same plate, using commercially available enzymatic reagents (Wako Chemicals, USA). Samples were read at A550 in a SpectraMax 384 Plus plate reader (Molecular Devices, Sunnyvale, CA).

### 2.3. Animal Procedures

Female wild type (WT) mice (C57BL/6N from Taconic, USA) and female homozygous apoC-II mutant mice, expressing a dysfunctional form of apoC-II [18], were fed a regular rodent chow diet (NIH31 chow diet; Zeigler Brothers Inc., Gardners, PA) but were fasted during the study. After a single IV bolus of injection of the lipid emulsion, retro-orbital blood collection was performed at the following time points: baseline, 5 min, 15 min, 30 min, 60 min, and 120 min. Blood samples were collected with heparinized capillary tubes (50 *µ*l) and placed into tubes with EDTA (final concentration 4 mM). Tubes were centrifuged at 1000 g for 20 minutes at 4°C to obtain plasma. After obtaining plasma, radioactivity was determined by a scintillation counter and expressed as counts per minute (CPM) in plasma (5 *µ*l) for each time point.

Mice were sacrificed at 120 min, placed on ice, perfused with cold saline, and organs were quickly removed and then weighed and stored at −70°C until analysis. Skeletal muscle was collected from the quadriceps, and white adipose tissue was collected from visceral fat in the peritoneal cavity. All animal studies were approved by the National Heart, Lung, and Blood Institute Animal Care and Use Committee (NIH Protocol #H-0050).

### 2.4. Lipid Extraction Assays

Lipids were extracted according to a previously published procedure [23]. Briefly, up to 150 mg piece of tissue was homogenized in methanol in a Dounce homogenizer, and lipids were extracted after more than a 72-hour incubation in a 2 : 1 (vol/vol) mixture of chloroform/methanol. The lower lipid layer was collected and counted for radioactivity by liquid scintillation. Results are expressed as CPM/tissue wet weight.

### 2.5. Plasma Measurements

The following fasting plasma lipids were enzymatically measured with kits from Wako Pure Chemicals (Osaka, Japan): total Cholesterol E for total cholesterol (TC), L-Type Triglyceride M for TG (glycerol blank method), and NEFA-HR (2) for FFAs.

### 2.6. Statistical Analyses

Unless otherwise indicated, all values are presented as mean ± SD for *in vitro* study and as mean ± SEM for *in vivo* studies. Results were analyzed with 2-way ANOVA, Student's *t*-test, and Tukey's multiple-comparison test. Plasma half-life of triolein, [9,10-^3^H(N)], was analyzed by a nonlinear Regression model, with the use of GraphPad Prism Software (San Diego, Calif). *P* < 0.05 was considered to be statistically significant.

## 3. Results

### 3.1. *In Vitro* Effect of C-II-a on LPL Lipolysis of Lipid Emulsion

**Figure 1:**
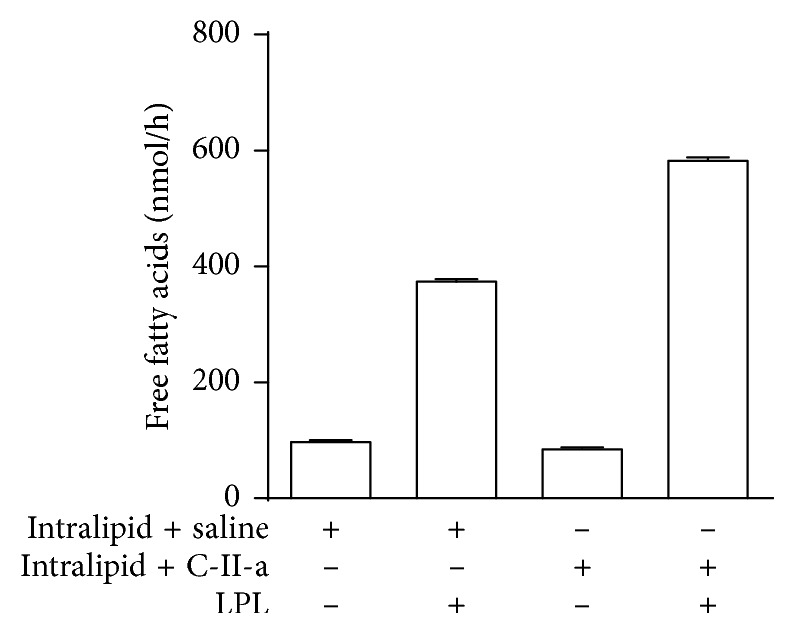
Effect of C-II-a on the *in vitro* lipolysis of TG. Free fatty acids released from the lipolysis of the TG-rich emulsion were monitored in the presence or absence of the peptide and LPL. Results represent the mean of triplicates ± S.D.

Incubation of the lipid emulsion with the C-II-a peptide did not change the mean size of the emulsion particles (approximately 300 nm) as measured by dynamic light scattering. It did, however, alter the ability of the lipid emulsion to undergo lipolysis by LPL (Figure 1). Purified LPL was added to the lipid-rich emulsion, with and without C-II-a, and the release of FFAs was measured. In the absence of LPL, the addition of the C-II-a peptide did not increase lipolysis over baseline, but there was nearly a 50% increase in the generation of FFAs after the addition of LPL compared to the vehicle control.

### 3.2. *In Vivo* Effect of C-II-a on Plasma Clearance of Lipid Emulsion

**Figure 2:**
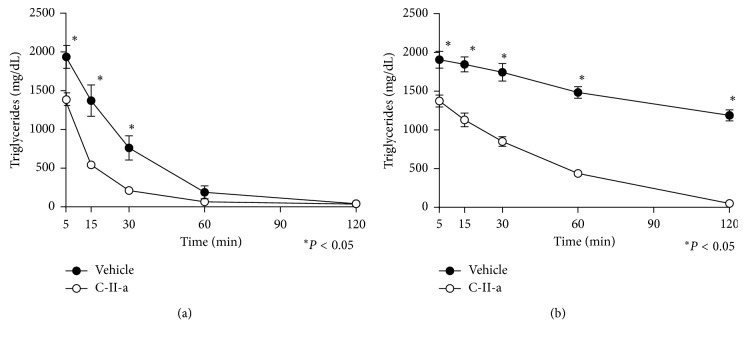
Effect of C-II-a on the *in vivo* clearance of plasma TG in C57BL/6N mice and apoC-II mutant mice. Results represent the mean ± S.E.M. of either C57BL/6N (*N*=3 per treatment group) (a) or apoC-II deficient mice (*N*=6 for peptide treated group vs *N* = 6 for vehicle group) (b). ^*∗*^*P* < 0.05.

To determine if the C-II-a peptide can also alter the *in vivo* plasma clearance of the lipid emulsion, we intravenously injected the lipid emulsion with and without the peptide into either WT (C57BL/6N) or apoC-II mutant mice (Figure 2). Typical baseline plasma lipid values of mice used in this study are shown in Tables 1 and 2.

For both lines of mice, we observed more rapid plasma clearance of TG in the presence of the C-II-a peptide. In case of WT mice, the peak TG level 5 min after injection was considerably higher than the 5 min time point observed for the mice treated with the peptide, which is consistent with the rapid action of the peptide we previously observed [17]. Thereafter, the rate of decline of plasma TG was similar with TG returning to near baseline values by 120 min, with or without the peptide. In case of the apoC-II-mutant mice, the clearance of TG from plasma was considerably slower compared to the C57BL/6N mice, with TG levels still over 1000 mg/dL at the 2 h time point. In contrast, plasma TG in the apoC-II mutant mice was more rapidly cleared after treatment with the C-II-a peptide, and TG returned to almost the same level as WT mice (<100 mg/dL) by the 2 h time point.

### 3.3. Distribution of Tissue Incorporation of Lipid Emulsion in C57BL/6N Mice

**Figure 3:**
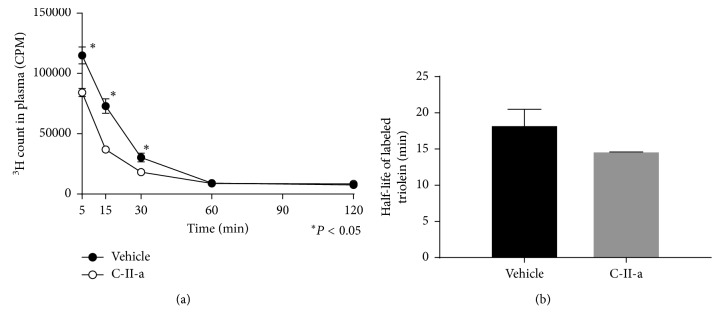
Effect of C-II-a on the *in vivo* clearance of plasma triolein radiotracer in wild type C57BL/6N mice. (a) Clearance of radioactive counts from plasma (5 *µ*l) at indicated time points. Results represent the mean of C-II-a (*N*=12) vs vehicle (*N*=9) ± S.E.M. (b) Calculated half-life of ^3^H-triolein tracer in plasma. ^*∗*^*P* < 0.05.

Next, we performed a similar type experiment as before in C57BL/6N mice but used instead a lipid emulsion in which radioactive triolein was incorporated into the particles, with the radioactive label in the fatty acids (Figure 3). Despite injecting identical amounts of lipid emulsion containing the triolein radiotracer into C57BL/6N mice, the first time point at 5 min was always consistently lower in the mice that received the lipid emulsion containing the C-II-a peptide. Thereafter, for both the vehicle control group and C-II-a treated mice, the radiolabeled triolein followed first-order exponential decay. The estimated half-life for the triolein in the C-II-a treated group was 14.8 ± 0.1 min, whereas the half-life for the vehicle control was longer (18.3 ± 2.2 min) (Figure 3), but the difference did not reach statistical significance (*P*=0.07). However, analysis of the individual time points showed a statistically significant reduction in the level of the TG radiotracer for the 5, 15, and 30 min time points when the peptide-treated group was compared to the control group that received the lipid emulsion without the peptide.

**Figure 4:**
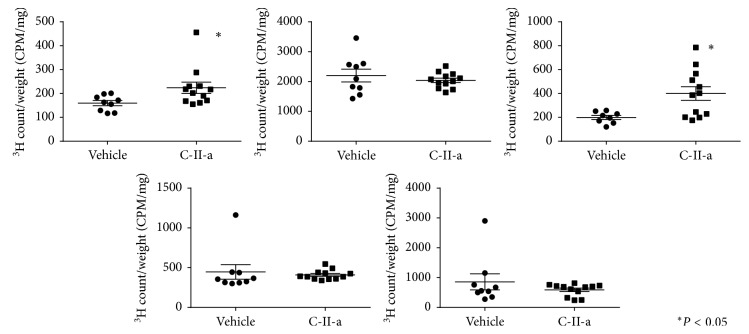
Effect of C-II-a on tissue incorporation of triolein radiotracer in organs of C57BL/6N mice. The data were expressed in CPM/mg (^3^H count/tissue weight). Results represent the mean of C-II-a (*N*=12) vs vehicle (*N*=9) ± S.E.M. ^*∗*^*P* < 0.05.

We next assessed the tissue distribution of the radiotracer (Figure 4). The presence of the C-II-a peptide on the lipid emulsion increased the incorporation of radioactive lipids by 1.4-fold in skeletal muscle (*P*=0.02) and 2-fold in visceral fat (*P*=0.01), respectively, the main organs known to take up TG [24]. Peptide treatment, however, did not have any apparent effect on incorporation of radiolabeled lipids into the liver, kidney, or spleen.

### 3.4. Distribution of Tissue Incorporation of Lipid Emulsion in ApoC-II Mutant Mice

**Figure 5:**
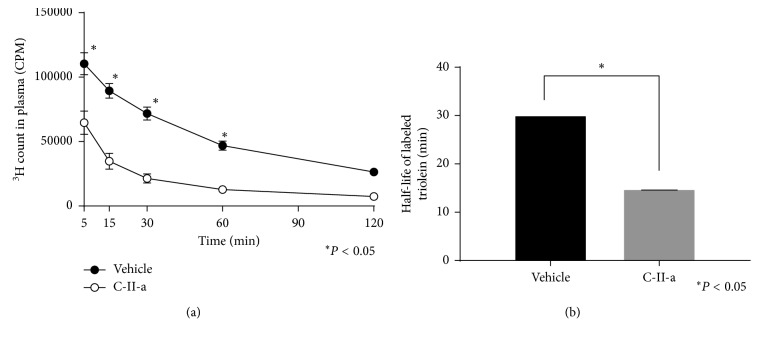
Effect of C-II-a on the *in vivo* clearance of plasma triolein radiotracer in apoC-II deficient mice. (a) Clearance of radioactive counts from plasma (5 *µ*l) at indicated time points. Results represent the mean of C-II-a (*N*=6) vs vehicle (*N*=6) ± S.E.M. (b) Calculated half-life of ^3^H-triolein tracer in plasma. ^*∗*^*P* < 0.05.

As observed in C57BL/6N mice, after injection of radiolabeled lipid emulsion, the first time point was consistently lower in the peptide-treated group versus the control, again consistent with the ability of the peptide to rapidly enhance plasma clearance of the TG-rich lipid emulsions (Figure 5). Also as before, the TG radiotracer showed an exponential decay in the apoC-II mutant mice but was considerably delayed versus the control C57BL/6N mice (vehicle, apoC-II mutant mice; *T*_1/2_ = 30.1 ± 0.1 min, C57BL/6N mice; *T*_1/2_ = 18.3 ± 2.2 min, and *P* < 0.001). The addition of the C-II-a peptide to the lipid emulsion considerably decreased the half-life of labeled triolein compared to the vehicle control group (apoC-II mutant mice, C-II-a; *T*_1/2_ = 14.8 ± 0.1 min, vehicle; *T*_1/2_ = 30.1 ± 0.1 min, *P* < 0.001). In fact, the half-life of the TG radiotracer in the apoC-II mutant mice was almost similar to that observed in the C57BL/6N mice after treatment with the peptide (C-II-a, apoC-II mutant mice; *T*_1/2_ = 14.8 ± 0.1 min, wild type mice; *T*_1/2_ = 14.7 ± 0.1 min, *P*=0.40, Figures 3 and 5).

**Figure 6:**
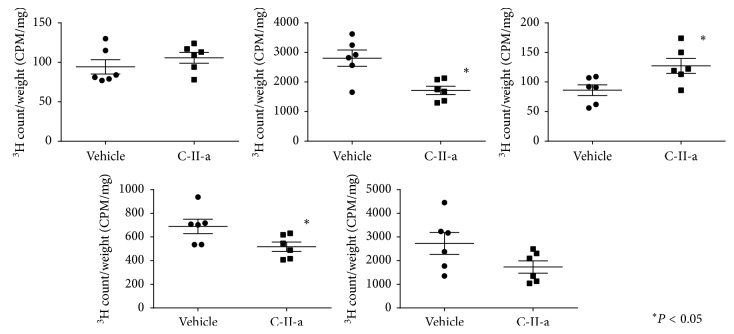
Effect of C-II-a on the tissue incorporation of triolein radiotracer in organs of apoC-II mutant mice. The data were expressed as CPM/mg (^3^H count/tissue weight). Results represent the mean of C-II-a (*N*=6) vs vehicle (*N*=6) ± S.E.M. ^*∗*^*P* < 0.05.

**Figure 7:**
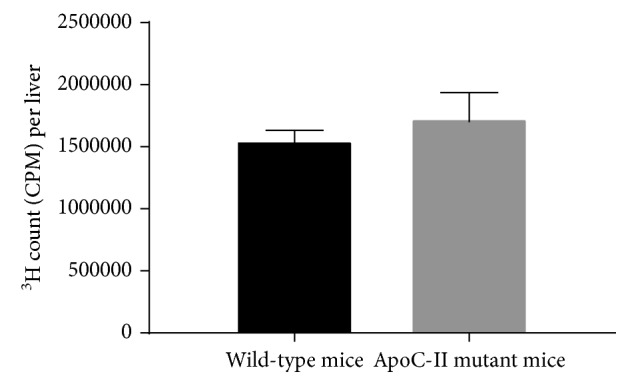
Effect of C-II-a on the hepatic incorporation of triolein radiotracer in wild-type C57BL/6N mice and apoC-II mutant mice. Results represent the mean for apoC-II mutant mice (*N*=6) vs wild-type C57BL/6 mice (*N*=12) ± S.E.M. ^*∗*^*P* < 0.05.

As we observed in C57BL/6N mice, apoC-II mutant mice injected with the radiolabeled lipid emulsion in the presence of the C-II-a peptide showed increased incorporation of the triolein tracer by 1.5-fold in visceral fat (*P*=0.02) (Figure 6). We also observed a trend for increased tracer incorporation in skeletal muscle, but this did not reach statistical significance unlike in C57BL/6N mice. Incorporation of radiolabeled lipids into the other tissues like the liver and kidney, however, appeared to decrease in the apoC-II mutant mice unlike what we observed in C57BL/6N mice. This could have possibly occurred because the more efficient lipolysis of TG and uptake of fatty acids into adipose tissue and skeletal muscle after the peptide treatment then lead to lower plasma TG levels and hence reduced hepatic uptake. This is consistent with the fact that the level of hepatic incorporation of the triolein tracer after the peptide treatment was nearly identical for the C57BL/6N mice and apoC-II mutant mice (wild type: 1549000 ± 83070 CPM, apoC-II mutant mice: 1728000 ± 207900 CPM, and *P*=0.35, Figure 7).

## 4. Discussion

HTG from apoC-II deficiency is an autosomal recessive disorder and thus it is widely believed that apoC-II is present in excess for what is normally needed to maximally activate LPL [1]. Some heterozygous family members with apoC-II deficiency, however, have been reported to have modest increases in TG [25, 26]. In our apoC-II mutant mice, we have also observed that heterozygous mice have increased plasma TG compared to their sibling wild type controls [18]. Furthermore, it has recently been reported that apoc2 gene is induced approximately 4-fold in the intestine of mice after gavage with a high fat meal [27]. Overall, these findings suggest that although apoC-II may normally be present in excess, under some physiologic conditions, it may become rate limiting for lipolysis. One of the main findings of this study is that endogenous apoC-II levels appear, in fact, to be insufficient for the rapid plasma clearance of TG when lipid-rich emulsions are intravenously infused similar to what is done for humans being treated with total parenteral nutrition.

The lipid emulsion used in this study, Intralipid, is derived from soybean oil and has a monolayer of phospholipids on the surface and TG in the core [28]. Because the particle size of the emulsion particle is comparable to that of chylomicrons and it rapidly acquires apolipoproteins, such as apoC-II, once it is infused into plasma, it is often used as an “artificial chylomicron” for studying TG metabolism. Intralipid was developed as a type of total parenteral nutrition for rapidly delivering energy in the form of TG into the tissues of undernourished patients [28]. Most patients after receiving Intralipid rapidly catabolize it within a few hours [28, 29], but in some patients it can persist, leading to a marked increase in plasma TG levels. In fact, infusion of Intralipid and other similar TG-rich emulsions have been reported to cause pancreatitis from HTG [30]. Another rare complication of Intralipid is acute respiratory distress syndrome, which may be due to the uptake of the TG-rich emulsion by pulmonary macrophages and perhaps by other tissue macrophages, causing the release of proinflammatory cytokines [31, 32].

We first showed *in vitro* that C-II-a, an apoC-II mimetic peptide, can activate LPL and increase the rate of Intralipid lipolysis. It did so without significantly altering the size of the lipid emulsion. The exact mechanism by which apoC-II or C-II-a activates LPL is not known but likely involves a protein-protein interaction with LPL. It may help LPL to anchor to surface of TG-rich particle despite the high surface tension created by the lipolysis of lipids [33]. When the radiolabeled lipid emulsion was associated with the C-II-a peptide and injected into C57BL/6N mice, it underwent more rapid plasma clearance compared to mice only treated with the vehicle. This is consistent with our *in vitro* observations and indicates that there is insufficient amount of endogenous apoC-II in wild type mice for maximal activation of LPL when a large load of TG is rapidly introduced into mice by intravenous injection. Besides activation by LPL, it is also possible that the peptide may have other mechanisms of action that may relate to the hepatic uptake of TG-rich lipoproteins, which is known to be critical in TG plasma clearance [8]. Because the dose of Intralipid used in this study was designed to match what is used in humans, apoC-II may also be rate limiting in TG plasma clearance of humans after treatment with total parenteral nutrition. As expected, the apoC-II mutant mice showed a marked decrease in plasma clearance of TG compared to wild type, but this was almost completely normalized after treatment with C-II-a peptide. This suggests that C-II-a or similar apoC-II mimetic peptides could potentially be a treatment for apoC-II deficiency. It is important to note that the apoC-II mutant mice used in this study were considerably older and heavier than the WT C57BL/6 mice (Tables 1 and 2). Thus, some of the differences in plasma TG clearance rate between the 2 strains of mice may be due to this difference, but it is unlikely to be a major factor and does not negate the clear effect of the C-II-a peptide in improving TG clearance in the apoC-II mutant mice.

Previous studies of soybean fat emulsions labeled with ^14^C-triolein have shown that the liver and adipose tissue are major sites of tissue uptake [21]. However, the lipolysis of TG and the tissue uptake of fatty acids undergo exquisite regulation and can quickly change depending on metabolic need. For example, adipose tissue continuously switches from being a TG storage organ in the fed state to releasing fatty acids in the fasted state by intracellular lipolysis [24]. This rapid switching occurs because of differential gene regulation of LPL and the ANGPTL family of proteins that can rapidly inhibit LPL [24]. The increased uptake in visceral fat observed in our study after the IV infusion of TG-rich emulsion is thus an expected outcome. The hydrolysis of TG and uptake of TG metabolites by skeletal muscle typically show the opposite pattern to that of adipose tissue. During the fed state, there is typically less fatty acid uptake in skeletal muscle but more uptake of glucose. We observed, however, increased incorporation of the radiotracer in skeletal muscle too after C-II-a treatment. This may have occurred because the mice were fasting, just prior to the lipid infusion, and thus likely still expressed higher levels of LPL in skeletal muscle, which was then activated by the apoC-II mimetic peptide. It is important to note, however, that the radiolabeled fatty acids in the triolein tracer can be converted to other metabolites by cells and released back into the general circulation, so the residual radioactive counts in the cells may not represent the total fatty acid uptake by the tissue. We tried to minimize this issue by quickly collecting the organs after sacrifice and storing them on ice, but for tissues with significant beta oxidation of fatty acids, such as skeletal muscle, the amount of total fatty acids initially taken up by the tissue may be underestimated from the radioactive counts. Despite this limitation, the difference observed in tissue incorporation of the radiotracer with and without the peptide treatment are still valid, but the precise quantitative impact on tissue uptake cannot be accurately determined.

In contrast to most tissues, the liver of adult mice does not express LPL and instead expresses a related protein called hepatic lipase, which does not require apoC-II for activation. This, therefore, could explain the lack of effect of C-II-a on increasing FFAs and TG metabolites uptake by the liver of wild type mice. Interestingly, in the apoC-II mutant mice, C-II-a treatment led to a significant decrease in hepatic incorporation of the triolein tracer. It is likely that the improved uptake stimulated by the peptide in the other tissues lowered plasma TG levels, which then resulted in less eventual uptake by the liver. In general, improving LPL lipolysis and increasing the uptake of fatty acids by peripheral tissues versus the liver is likely to be beneficial. Excess fatty acid delivery to the liver, which commonly occurs in metabolic syndrome, causes increased VLDL production and eventually fatty liver disease and hepatic insulin resistance [34]. In contrast, fatty acids that are delivered to skeletal muscle are largely used for energy production and also stimulate the biogenesis of mitochondria, which may then dissipate excess energy by generating heat by uncoupling oxidative phosphorylation [35]. Transgenic mice expressing excess LPL in skeletal muscle, however, eventually develop lipotoxicity and insulin resistance [36, 37]. The observation in this study that the C-II-a peptide can alter the tissue uptake of TG-rich emulsion warrants further investigation because this could perhaps be used therapeutically, particularly when applied in conjunction with a low-fat diet.

In conclusion, C-II-a, an apoC-II mimetic peptide, was shown to increase the plasma clearance of TG-rich emulsions in mice, thus indicating that endogenous apoC-II may be rate limiting under certain conditions, such as during total parenteral nutrition. Furthermore, C-II-a was shown to differentially alter the incorporation of TG from lipid-rich emulsions into peripheral tissue and the liver. Much more work will be needed to extend these studies to humans, but the results suggest that apoC-II mimetic peptides could offer a new therapeutic strategy for favorably improving the disposition of TG after lipid-rich emulsion infusion and for avoiding some of the rare adverse consequences of this type of nutritional therapy. ApoC-II mimetic peptides could also possibly be beneficial in some metabolic diseases, such as in diabetes, in which delivery of TG by lipoproteins to peripheral tissues is known to be impaired [38].

## Figures and Tables

**Table 1:** Characteristics of wild-type mice.

	Vehicle (*N*=9)	C-II-a (*N*=12)
Number	9	12
Age (Mo)	2.3 ± 0.0	2.3 ± 0.0
Body weight (g)	18.2 ± 0.2	18.9 ± 0.3
TG (mg/dL)	88.9 ± 3.5	89.0 ± 2.9
FFAs (mmol/L)	0.3 ± 0.1	0.3 ± 0.1
TC (mg/dL)	76.2 ± 2.1	76.7 ± 2.0

Vehicle = intralipid + saline; C-II-a = intralipid + apoC-II active peptide. All mice were females on a regular chow diet. Results represent the mean of duplicate ± S.E.M.

**Table 2:** Characteristics of apoC-II mutant mice.

	Vehicle (*N*=6)	C-II-a (*N*=6)
Number	6	6
Age (Mo)	11.6 ± 1.1	11.5 ± 0.6
Body weight (g)	32.0 ± 2.4	32.0 ± 2.8
TG (mg/dL)	716.5 ± 102.1	787.4 ± 106.1
FFAs (mmol/L)	3.0 ± 0.1	2.9 ± 0.3
TC (mg/dL)	65.3 ± 6.0	68.8 ± 6.5

Vehicle = intralipid + saline; C-II-a = intralipid + apoC-II active peptide. All mice were females on a regular chow diet. Results represent the mean of duplicate ± S.E.M.

## Data Availability

The research data used to support the findings of this study are included within the article.
